# Editorial: Inflammation and oxidative stress in vascular and renal disease: role of the inflammasome and pyroptosis

**DOI:** 10.3389/fphys.2023.1270554

**Published:** 2023-09-05

**Authors:** Carmen De Miguel, Rodrigo O. Marañón, Santiago Cuevas

**Affiliations:** ^1^ Section of Cardio-Renal Physiology and Medicine, Division of Nephrology, Department of Medicine, Heersink School of Medicine, University of Alabama at Birmingham, Birmingham, AL, United States; ^2^ Department of Morphophysiology, Faculty of Medicine, Institute of Physiology, High Institute of Biological Science (INSIBIO) and Centro Científico Tecnológico—Consejo Nacional de Investigaciones Científicas y Técnicas (CCT CONICET) Tucumán, National University of Tucumán, Tucumán, Argentina; ^3^ Biomedical Research Institute of Murcia (IMIB), Palmar, Spain

**Keywords:** inflammation, oxidative stress, vascular disease, renal disease, inflammasome, pyroptosis

## Introduction

Inflammation and reactive oxygen species (ROS) production are complex processes that involve local and systemic responses to a variety of different stimuli. Dysregulation of these processes is characteristic in the pathogenesis of cardio-renal diseases. Despite the numerous publications and efforts dedicated to better understanding this issue in the last 20 years, our knowledge of the complex interplay between inflammation, oxidative stress, and redox balance in vascular and renal pathology remains scarce. In this context, recent reports highlight the critical role of the inflammasome in regulating sterile inflammation, and identify this complex as a possible key target for attenuating inflammation during cardio-renal pathologies. The articles published within this Research Topic, “*Inflammation and oxidative stress in vascular and renal disease: role of the inflammasome and pyroptosis*” explore the redox physiology underlying these diseases, and shed light on the role of the inflammasome and pyroptosis in their progression. This editorial provides an overview of the Research Topic, highlighting its significance and summarizes the contributions made by the five included articles: four original articles and one review article.

## Significance of the Research Topic

Cardiovascular diseases are the leading cause of death worldwide, causing more than 17.9 million deaths in 2019, according to the World Health Organization. In particular, vascular and renal diseases, including hypertension, atherosclerosis, diabetic nephropathy, and chronic kidney disease, pose significant health challenges worldwide. Although inflammation and oxidative stress are critical and common factors in the development and progression of these conditions, we are far from having a complete understanding of the molecular mechanisms involved. This lack of understanding of how inflammation and oxidative stress contribute to vascular and renal pathology hinders the development of effective therapies for the treatment of the affected patients. This Research Topic highlights the implication of different novel pathways, from molecular mechanisms of inflammasome activation to monocyte-derived extracellular vesicles, sex-dependent pathways, and new biomarkers of oxidative stress in these diseases. Here, we briefly introduce and review the highlights of each article’s contribution in the following section.

## Contributions of the Research Topic

In this Research Topic, Ertuglu et al. authored a comprehensive review of the current literature regarding the role of inflammation and oxidative stress in salt-sensitive hypertension, emphasizing the role of NLRP3 inflammasome activation and discussing potential therapeutic approaches to this disease. This review paper offers valuable insights into this emerging area of research, providing updated evidence on genetic polymorphisms in NLRP3 as well as IL-1β genes associated with hypertension in humans while also reviewing evidence of activation of the inflammasome in animal models of salt-sensitive hypertension. The authors also review the NLRP3 inflammasome’s implication in the development of vascular dysfunction during hypertension, as well as recent evidence that suggests that oxidative stress triggers the activation of this inflammasome. Moreover, this review also highlights the pro-inflammatory effect of isolevuglandins (IsoLGs), lipid-derived protein modifications that have been recently associated with increased sodium reabsorption in the kidney and have an important role on T cell activation.

The first original paper in this Research Topic covers the topic of sex differences in the mechanisms involved in blood pressure regulation. Using the spontaneously hypertensive rat (SHR), a well-established animal model of essential hypertension, Abdelbary et al. investigated the intriguing hypothesis that differences in apoptosis drive the decreased blood pressure and kidney inflammatory response that is common in female SHRs. After using a pan-caspase inhibitor to decrease apoptosis in both sexes, the authors observed similar decreases in renal apoptosis in male and female rats, but no alterations in the blood pressure or renal T cell profiles. Therefore, they concluded that sex differences in apoptosis do not contribute to sex differences in blood pressure or renal T cell infiltration during essential hypertension.

In the second original paper, Pitzer Mutchler et al. studied the implications of Mg^2+^ deficiency in the pathogenesis of hypertension. In particular, the authors investigated the interplay between Mg^2+^ deficiency, NLRP3 inflammasome activation in monocytes and dendritic cells, and the production of IsoLGs by these cells. As mentioned above, IsoLGs are thought to play a critical role in activating the inflammatory cascade during hypertension. By using a combination of *in vivo* and *in vitro* approaches, the authors demonstrate that depletion of dietary Mg^2+^ results in increased blood pressure levels, as well as exaggerated activation of the NLRP3 inflammasome and IL-1β production. These findings may have important clinical implications since commonly used medications like diuretics lead to a deficiency of Mg^2+^.

Preeclampsia, or new onset hypertension developed after the 20th week of gestation, is a significant complication of pregnancy, making the identification of potential therapeutic interventions of great clinical importance. Preeclampsia is characterized by excessive inflammation, endothelial dysfunction, and end-organ damage (Rana et al., 2019), as well as elevated circulating levels of microvesicles. In their original paper, Santoyo et al. unveiled the potential role of pravastatin in reducing plasma levels of extracellular vesicles in women at risk for preeclampsia. Their findings suggest that pravastatin, commonly used for reducing high cholesterol, may prevent the endothelial dysfunction typical of this disease, highlighting this drug as a potential preventative therapy in the clinic.

Finally, Pei et al. performed an in-depth study of potential oxidative stress biomarkers associated with renal ischemia-reperfusion injury and their relationship with kidney inflammation in these conditions. By doing a comprehensive analysis of differentially expressed genes (DEGs) during oxidative stress and inflammation, they identified glutathione peroxidase 3 (GPX3) and glutathione S-transferase 1 (GSTT1) were significantly positively correlated with plasma cells and macrophage M0, while were negatively correlated with monocytes and macrophages M1 and M2. Overall, the manuscript presents valuable insights into the pathophysiological mechanisms underlying renal ischemia-reperfusion injury and suggests that GPX3 and GSTT1 might play a key role in the physiological and pathological processes involved. The authors also underline the potential utility of GPX3 and GSTT1 as potential therapeutic targets and biomarkers for diagnosing renal ischemia-reperfusion injury.

## Conclusion

In summary, this Research Topic presents a collection of diverse articles that expand our understanding of redox physiology during vascular and renal diseases. This Research Topic provides an overview of the current advances in investigation in this field, shedding light on the intricate interplay between inflammation and oxidative stress and providing valuable insights into the underlying mechanisms and potential novel therapeutic targets against cardio-renal disease.
